# Ergotamine Targets KIF5A to Facilitate Anoikis in Lung Adenocarcinoma

**DOI:** 10.1111/crj.70020

**Published:** 2024-11-08

**Authors:** Bin Bao, Xiaojun Yu, Wujun Zheng, Jiewei Sun

**Affiliations:** ^1^ Cardiothoracic Surgery Department The First People's Hospital of Fuyang Hangzhou China

**Keywords:** anoikis, Ergotamine, kinesin family member 5A, lung adenocarcinoma

## Abstract

**Background:**

Kinesin family member 5A (KIF5A) has been reported to be closely related to cancer progression. The aim of this study was to investigate the effect of KIF5A on lung adenocarcinoma (LUAD) and its potential molecular mechanisms.

**Methods:**

Using bioinformatics analysis methods and molecular experiments, the expression of KIF5A in LUAD was analyzed, with its expression in attached and detached tumor cells assessed. Gene set enrichment analysis (GSEA) of KIF5A was carried out. The small molecular drug with the highest affinity for KIF5A was screened out through molecular docking experiments, which was validated through cellular thermal shift assay (CETSA). Quantitative polymerase chain reaction (qPCR) was employed to measure the expression levels of anoikis‐repressing genes (BCL2, CAV1), as well as anoikis‐inducing gene (PDCD4). CCK‐8 assay was applied to examine cell viability. Cell colony formation experiments were utilized to evaluate cell proliferation ability.

**Results:**

We observed that KIF5A was highly upregulated in LUAD tissues and cells, with a higher level detected in detached LUAD cells. By resorting to bioinformatics analysis, we discovered that KIF5A was abundant in the anoikis pathway. Knocking down KIF5A reinforced anoikis in LUAD. Further screening identified Ergotamine as the small molecular drug with the highest affinity for KIF5A. The CETSA confirmed the binding relationship between the two. In addition, Ergotamine has a promoting effect on the anoikis of LUAD, while overexpression of KIF5A reversed the effects of Ergotamine on LUAD cells.

**Conclusion:**

This project uncovered that the small molecular drug Ergotamine targets and inhibits the expression of KIF5A. Downregulated KIF5A can enhance the anoikis of LUAD. Our results supported the inhibition of KIF5A as an attractive therapeutic strategy for LUAD. This finding provides a new innovative pathway for the treatment of LUAD and offers a strong theoretical basis for the development of therapeutic drugs targeting KIF5A.

## Graphical abstract

Kinesin family member 5A (KIF5A) inhibits lung adenocarcinoma (LUAD) cell anoikis, while the small molecule drug Ergotamine targets and inhibits the expression of KIF5A, which in turn induces LUAD cell anoikis.

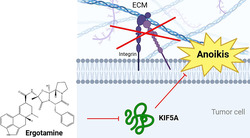

## 1 Introduction

Lung cancer (LC) remains a daunting challenge facing human beings, standing as the leading culprit of cancer‐related deaths worldwide [1]. Among them, nonsmall cell lung cancer (NSCLC) accounts for 80% to 85%, with lung adenocarcinoma (LUAD) as the most common histological type of NSCLC and exhibiting the characteristics of strong aggressiveness, early metastasis, and poor prognosis [2]. Although treatment for LUAD has made great progress in recent years, patients still have a low 5‐year survival rate [3, 4, 5]. Therefore, innovating new therapeutic targets and novel drugs for LUAD treatment is an urgent need.

Anoikis is a form of anchorage‐dependent programmed cell death, which refers to apoptosis caused by loss of adhesion between cells and neighboring cells or extracellular matrices [6]. A large body of studies has pointed out that anoikis plays an instrumental part in repressing the survival and reattachment of free tumor cells to new matrices, functioning as a barrier to tumor metastasis [7]. However, anoikis resistance occurs in malignant tumors, including LUAD, which is critical to tumor progression and metastasis. For instance, Lim et al. [8] discovered that polysaccharides derived from persimmon leaves have the promoting effect on anoikis of LC cell A549 as well as the repressing impact on cell migration, invasion, and epithelial–mesenchymal transition (EMT). A large number of genes related to anoikis have been identified to predict LUAD prognosis and immune status [9, 10, 11]. Additionally, extracellular matrix (ECM), cell adhesion, regulatory proteins, cytoskeletal regulators, cell detachment, and targeted migration are all implicated in the regulation of anoikis in LC cells [12]. Cavelin‐1 (CAV1) is a key protein that boosts the directional migration of cells. CAV1 is found to repress cell anoikis. In a study on LUAD, the expression of CAV1 was suppressed by miR‐1827, and downregulated CAV1 induced anoikis in LUAD cells [13]. B‐cell lymphoma‐2 (BCL2) is a mitochondria‐localized protein that plays a role in the regulation of apoptosis [14]. BCL2 inhibitor of transcription 1 (Bit1) is named for its ability to reduce the activity of the BCL2 promoter. After cell adhesion mediated by integrins or the ECM, Bit1 is released from the mitochondria to the cytoplasm, where it forms a complex with the transcriptional regulatory factor amino‐terminal enhancer split (AES), inducing cell anoikis [12, 15]. BCL2 expression can be enhanced by Bone Morphogenetic Protein 4 (BMP4), which promotes anoikis resistance in breast cancer cells [16]. In addition, programmed cell death 4 (PDCD4) is a tumor suppressor that induces apoptosis [17]. It was found that PDCD4 expression was downregulated after targeted inhibition by miR‐21, which suppressed anoikis in human esophageal adenocarcinoma [18]. The relationship of anoikis resistance with tumor cell survival and metastasis has received a lot of attention. Therefore, further investigation of the molecular mechanisms that regulate anoikis in LUAD cells is critical for exploring more potential targets to facilitate anoikis.

Kinesin family member 5A (KIF5A) is one of the subtypes of the kinesin family‐1 (Kinesin, also known as KIF5), making up three subtypes of KIF5 in mammals along with KIF5B and KIF5C [19, 20]. KIF5A is a neuron‐specific cytoskeletal protein that can participate in the transport of various macromolecules such as ribonucleoproteins, featuring in the modulation of biological processes such as cell division, proliferation, and differentiation [21]. KIF5A, a member of the kinesin motors family, is able to transport RNP particles, vesicles, and other organelles in a retrograde direction to axon terminals [22]. Research has found that KIF5A is responsible for the transport and secretion of the ECM protein collagen‐1 [23]. Currently, KIF5A is extensively studied in the field of neurodegenerative diseases. Notably, KIF5A, however, is also tightly linked to the occurrence and progression of various human cancers. The high expression of KIF5A is reported to be connected to the dismal prognosis of bladder cancer (BCa) [24], breast cancer (BC) [25], and prostate cancer (PCa) [26]. In addition, by using SELDI‐TOF and CART technologies, Tooker et al. [27] revealed KIF5A as a blood biomarker to identify asbestosis patients with LC risk. However, there is no work evaluating the expression and function of KIF5A in LUAD yet. Therefore, it is necessary to explore the role of KIF5A on LUAD and its underlying molecular mechanisms, which may facilitate the development of personalized therapeutic strategies for LUAD.

In this study, we demonstrated that KIF5A was highly upregulated in LUAD, exhibiting the inhibiting impact on LUAD cells as well as the promoting impact on tumor cell proliferation. Moreover, we identified the binding relationship between Ergotamine and KIF5A. Further research revealed that Ergotamine targeted and bonded to KIF5A, repressing its expression and reinforcing LUAD cell anoikis. Our results revealed the critical roles of Ergotamine and KIF5A in anoikis of LUAD, indicating that Ergotamine may be an effective inhibitor targeting KIF5A.

## 2 Materials and Methods

### 2.1 Bioinformatics Analysis

mRNA expression data of LUAD patients were available for download at The Cancer Genome Atlas (TCGA) database (https://portal.gdc.cancer.gov/), comprising 539 tumor tissues and 59 normal lung tissues (from nontumor patients). We carried out the differential analysis on the data of tumor and normal groups (|logFC| > 1.0, FDR < 0.05) to obtain differentially expressed mRNA (DEmRNA), with the target gene KIF5A identified based on referencing literature. Gene set enrichment analysis (GSEA) was launched on the target gene to identify the signaling pathways in which it was enriched. Survival analysis of the target gene was performed using Kaplan–Meier Plotter (http://kmplot.com/analysis/).

By using small molecules approved by the Food and Drug Administration (FDA) as ligands, we performed batch molecular docking on target genes. The predicted protein PDB structures of target genes were downloaded from the Uniprot database (https://www.uniprot.org/), with receptor pockets predicted by ProteinsPlus (https://proteins.plus/). The pocket 0 was selected. The AutoDock was utilized to set up the box and wrap the pocket. The box size and coordinate parameters were recorded. We downloaded the FDA mol2 files from the network (https://zinc.docking.org/substances/subsets/fda/), split files by utilizing OpenBabe software, and obtained individual small molecule mol2 files. We employed MGLTools software to convert the mol2 files into pdbqt files and performed batch molecular docking.

### 2.2 Cell Cultivation

Human bronchial epithelial cell line BEAS‐2B (BNCC359274) and human‐derived LUAD cell lines NCI‐H2087 (BNCC100691) and A549 (BNCC337696) were all bought from BeNa Culture Collection (China). We cultivated BEAS‐2B cells in a DMEM‐H medium (Gibco, USA) and A549 cells in an F‐12 K medium (Gibco, USA), both of which contained 10% fetal bovine serum (FBS) and 1% streptomycin/penicillin. NCI‐H2087 cells were cultured in a DMEM/F‐12 medium (Gibco, USA) containing 10% FBS, 50‐nM hydrocortisone, 1% ITS, 1 ng/mL EGF, 10‐mM HEPES, 4‐mM sodium pyruvate, and 2‐mM L‐glutamine. Finally, the cells were kept in a humid environment at 37°C with 5% CO_2_.

### 2.3 Suspension Culture

After trypsin digestion, cells during the logarithmic growth were collected, with 4 × 10^5^ cells inoculated on an ultralow adsorption cell culture plate. They were housed in an incubator containing 5% CO_2_ at 37°C and suspended for 48 h to simulate in vitro anoikis status of LUAD cells that were detached from the ECM and surrounding cells.

### 2.4 Cell Transfection and Drug Treatment

pcDNA3.1‐KIF5A expressing plasmid (oe‐KIF5A), pLKO.1‐KIF5A expressing plasmid (sh‐KIF5A), and corresponding negative controls were all purchased from RiboBio (China). Plasmids were transfected into the corresponding cells using Lipofectamine 2000 (Thermo Fisher Scientific, USA) and incubated for 48 h for subsequent experiments. LUAD cells were plated in a six‐well plate, with 20 μL of virus solution and polybrene (5 μg/mL) introduced after 24 h. Another 24 h after infection, we replaced the cell culture medium containing the virus solution with a fresh complete one containing Puromycin (2 μg/mL). The LUAD cells were treated with 10‐μM Ergotamine for 48 h, readying for subsequent assessment [28].

### 2.5 Quantitative Polymerase Chain Reaction (qPCR)

Total RNA of cells was extracted using RNAiso Plus (TAKARA, Japan), with RNA concentration and purity measured by NanoDROP ND‐1000 spectrophotometer (Thermo Fisher Scientific, USA). RNA was reversely transcribed into cDNA using PrimeScript™ RT reagent kit (TAKARA, Japan). qPCR was carried out by utilizing TB Green® Premix Ex Taq™ II (TAKARA, Japan) on an ABI 7500 PCR system (Applied Biosystems, USA) for qRT‐PCR analysis, with GAPDH as the reference. The relative mRNA expression levels were calculated using the 2^‐ΔΔCt^ method. Table 1 lists the primers in qPCR.

**TABLE 1:** Primers for qRT‐PCR.

Gene	Primer sequence (5′ → 3′)
KIF5A	Forward primer:GAGAACGATGCCGCTAAGGAT
	Reverse primer:TGCCCACAATGACACTGAACT
BCL2	Forward primer:AGGCTGGGATGCCTTTGTGGAA
	Reverse primer:ACCAGGGCCAAACTGAGCAGA
CAV1	Forward primer:GCGACCCTAAACACCTCAAC
	Reverse primer:ATGCCGTGTCAAACTGTGTGTC
PDCD4	Forward primer:TGGATTAACTGTGCCAACCA
	Reverse primer:TCTCAAATGCCCTTTCATCC
GAPDH	Forward primer:AAGGTCGGAGTCAACGGATTTGG
	Reverse primer:TTCTCAGCCTTGACGGTGCC

### 2.6 CCK‐8 Assay

Every 2 × 10^3^ cells were plated in a well of a 96‐well cell culture plate. After the cells adhered, 10 μL of CCK‐8 reagent (Beyotime, China) was introduced to each well at 0, 24, 48, and 72 h of culture. We incubated cells at 37°C in a light‐avoiding environment for 2 h and measured the absorbance at 450 nm by placing the plate on the microplate reader.

We seeded cells in a 96‐well cell culture plate (density: 5 × 10^3^ cells/well). The cells were treated with a gradient concentration of Ergotamine (0, 2.5, 5, 7.5, 10 μM) for 48 h, followed by the addition of 10 μL of CCK‐8 reagent (Beyotime, China) into each well. The cells were kept at 37°C in a light‐avoiding environment for 2 h. The absorbance at 450 nm was measured by placing the plate on the microplate reader.

### 2.7 Colony Formation Assay

Cell suspensions from each group were collected and seeded into a 12‐well plate at a density of 200 cells/well. We cultivated them in a fresh medium for 14 days, fixed them with 4% paraformaldehyde, and stained them with 0.1% crystal violet solution (Solarbio, China). The cells were rinsed several times with phosphate‐buffered saline (PBS), dried, and photographed with a digital camera.

### 2.8 Western Blot (WB)

Cells from different treatment groups were collected and lysed with RIPA lysis buffer (Beyotime, China) that contained protease and phosphatase inhibitors. Total protein was extracted, with protein concentration assessed by utilizing the BCA protein assay kit (Beyotime, China). Protein samples were isolated by using 10% SDS‐PAGE and were transferred to the PVDF membrane (Millipore, USA). The membrane was blocked with 5% skim milk at room temperature for 1 h. After 1:1000 dilution, we introduced primary rabbit antihuman KIF5A antibody and GAPDH antibody (Abcam, UK) to the samples and incubated them overnight at 4°C. After TBST rinse, we added the secondary antibody horseradish peroxidase (HRP)‐labeled Goat Anti‐Rabbit IgG (H + L) (Abcam, UK) for 1 h of incubation at room temperature. After rewashing with TBST, the Omni‐ECL™ ultrasensitive chemiluminescence kit (Epizyme Biotech, China) was employed to develop color and take band images through the ChemiScope 6000 chemiluminescence imaging system (Clinx, China).

### 2.9 Cellular Thermal Shift Assay (CETSA)

After treating cells with Ergotamine (10 μM) or DMSO for 24 h, we gathered the cells, lysed them with a lysis buffer containing protease inhibitors, and distributed the lysate evenly into 6 PCR tubes, which were heated at 37°C, 42°C, 47°C, 52°C, 57°C, and 62°C for 3 min to denature the proteins and were then rapidly frozen twice in liquid nitrogen. After centrifugation (12 000 rpm, 4°C, 20 min), we collected the supernatant and added an equal amount of 2 × SDS loading buffer to the supernatant, with the protein quantified by using WB.

### 2.10 Transwell Assay

For migration assay experiments, transfected cells (2 × 10 [4]/well) were resuspended with serum‐free medium and added to the upper chamber (Becton Dickinson, USA), and then medium containing 10% FBS was added to the lower chamber, and after 24 h of incubation, the bottom cells were fixed in methanol for 30 min and then stained with 0.1% crystal violet and photographed to record the cell migration.

For the invasion assay experiments, cells (2 × 10^4^ cells/well) were inoculated in the upper chamber of serum‐free medium coated with matrix gel, and the rest of the steps were the same as those for the migration experiments.

### 2.11 Data Statistics and Analysis

The results of assays were analyzed and plotted using GraphPad Prism 8.0 software. All experiments were independently repeated at least three times and presented as mean ± SD. The comparison between the two groups was achieved by utilizing the *t*‐test. The comparison among multiple groups was achieved by using the one‐way analysis of variance (ANOVA). *p* < 0.05 is deemed statistically significant.

## 3 Results

### 3.1 Upregulation of KIF5A Gene Expression in LUAD

**FIGURE 1:**
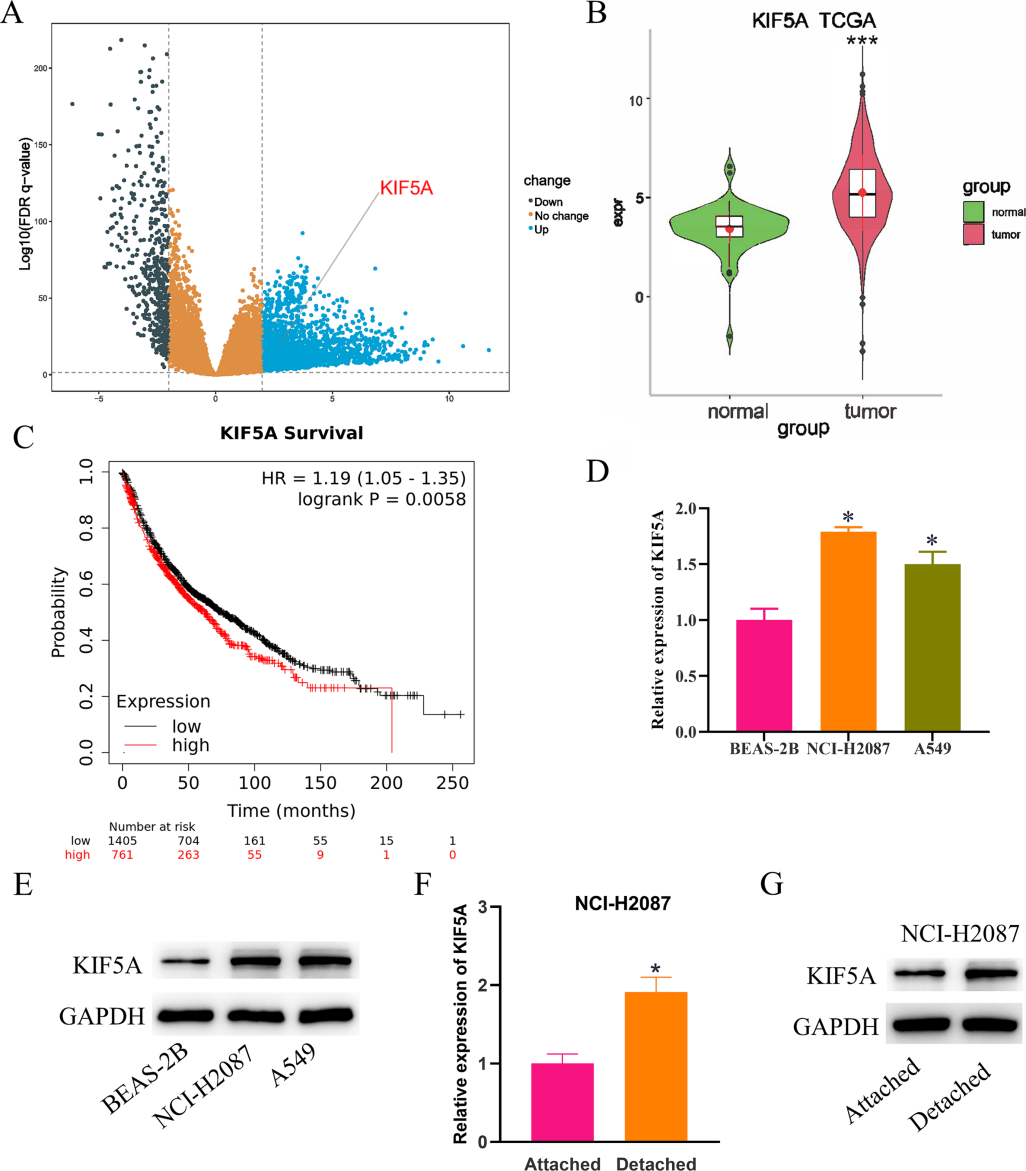
Upregulation of KIF5A in LUAD. (A) Differentially expressed genes in LUAD as analyzed using the TCGA database. (B) The analysis of KIF5A expression in LUAD tumor tissues and adjacent normal tissues using the TCGA database. (C) Survival analysis showed the relationship between KIF5A expression in LUAD tumors and patient prognosis. (D,E) qPCR and WB detected KIF5A expression in normal human bronchial epithelial cells (BEAS‐2B) and human LUAD cell lines (NCI‐H2087, A549). (F,G) NCI‐H2087 cells were divided into attached and detached groups, with KIF5A expression in different treatment groups measured by qPCR and WB. * means *p* < 0.05.

We first detected differentially expressed genes in LUAD by using the TCGA database (Figure 1A and Table S1). Next, we measured the differential expression of KIF5A in LUAD tumor tissues and adjacent normal tissues, finding that the expression of KIF5A was considerably upregulated in LUAD tissues (Figure 1B). We also found that high KIF5A expression was significantly associated with poor prognosis in LUAD patients using survival curve analysis (Figure 1C). In addition, we assessed the expression of KIF5A in normal human bronchial epithelial cell line (BEAS‐2B) and human LUAD cell lines (NCI‐H2087, A549) by utilizing qPCR and WB, which demonstrated that compared to normal human bronchial epithelial cells, the expression of KIF5A was also upregulated in LUAD cells (Figure 1D,E). Subsequently, we inoculated NCI‐H2087 cells on ultralow attachment plates and normal attachment plates to set up attached and detached groups of LUAD cells. qPCR results uncovered that compared to the attached group, the expression level of KIF5A was increased in detached NCI‐H2087 cells (Figure 1F). The WB detection obtained consistent results with qPCR (Figure 1G). Taken together, KIF5A is highly expressed in LUAD tissues and cells, with a higher level observed in detached LUAD cells.

### 3.2 KIF5A Represses Anoikis in LUAD Cells

**FIGURE 2:**
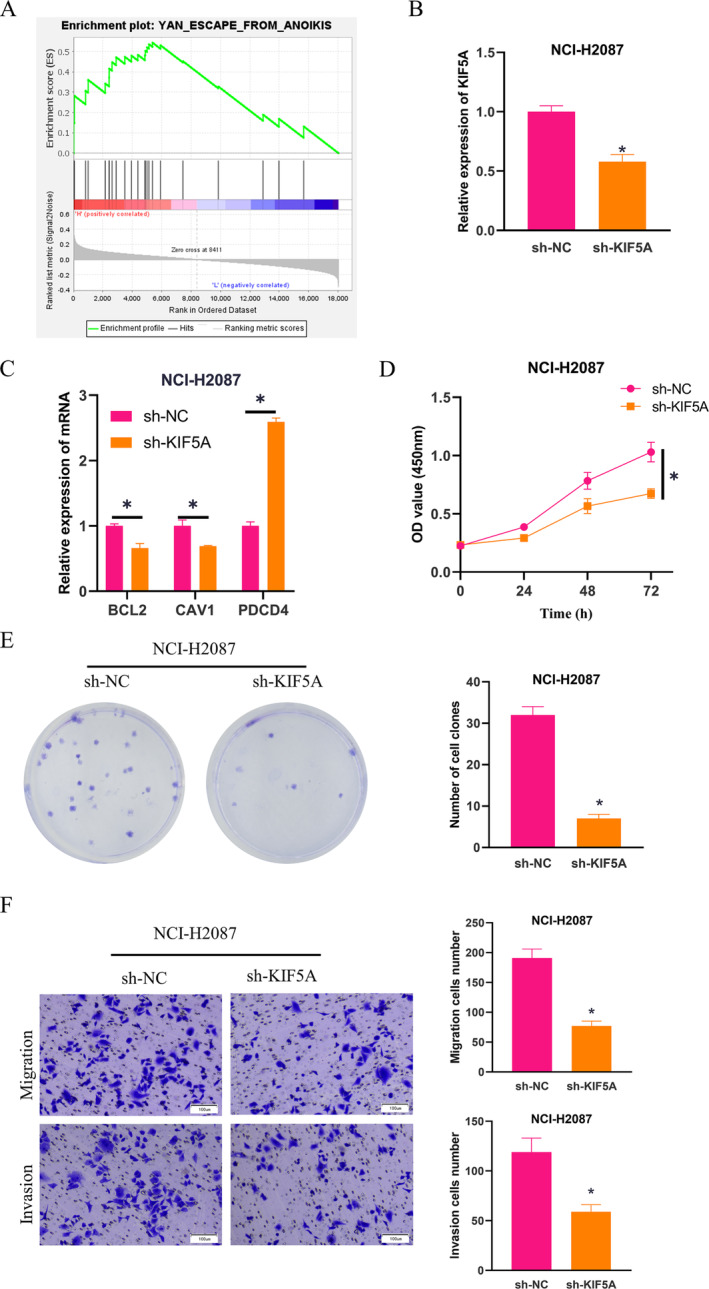
The effect of KIF5A on anoikis of LUAD cells. (A) Signaling pathways enriched by KIF5A analyzed by GSEA. (B) qPCR assessed KIF5A expression in different groups of NCI‐H2087 cells. (C) qPCR measured the expression levels of anoikis‐repressing genes (BCL2 and CAV1) and the anoikis‐inducing gene PDCD4 in different groups of LUAD cells. (D) CCK‐8 assay determined the viability of NCI‐H2087 cells in different groups. (E) Cell colony formation experiment measured the proliferation ability of NCI‐H2087 cells in different groups. (F) Transwell assay measured the migration and invasion abilities of NCI‐H2087 cells in different groups. * means *p* < 0.05.

Through analysis conducted by bioinformatics software, we uncovered that KIF5A was enriched in the anoikis signaling pathway (Figure 2A). To further probe into the impact of KIF5A on LUAD anoikis, we constructed cell groups (sh‐NC, sh‐KIF5A) based on NCI‐H2087 cells and inoculated the cells on ultralow attachment plates to prevent cell adhesion, making cells at a suspended status. First, the expression of KIF5A in different groups of NCI‐H2087 cells was examined by qPCR, which revealed that knocking down KIF5A considerably reduced the expression level of KIF5A in suspended cells, suggesting good transfection efficiency (Figure 2B). Subsequently, we employed qPCR to measure the expression of anoikis‐repressing genes (BCL2 and CAV1) as well as anoikis‐inducing gene PDCD4 in different groups of suspended LUAD cells, unearthing that compared to the control group, knocking down KIF5A strikingly suppressed the expression of BCL2 and CAV1 in NCI‐H2087 cells but increased the expression of PDCD4 (Figure 2C). CCK‐8 was carried out to determine the viability of suspended NCI‐H2087 cells in different groups, revealing that knocking down KIF5A strikingly dampened the vitality of tumor cells (Figure 2D). Next, the proliferation ability of suspended NCI‐H2087 cells was evaluated using a cell colony formation experiment, which demonstrated that knocking down KIF5A reduced the number of colonies formed by NCI‐H2087 cells (Figure 2E). We also found by Transwell assay that knockdown of KIF5A resulted in a significant reduction in the migration and invasion ability of NCI‐H2087 cells (Figure 2F). Our results suggested that KIF5A can suppress anoikis in LUAD cells.

### 3.3 Ergotamine Binds to KIF5A to Enhance Anoikis in LUAD

**FIGURE 3:**
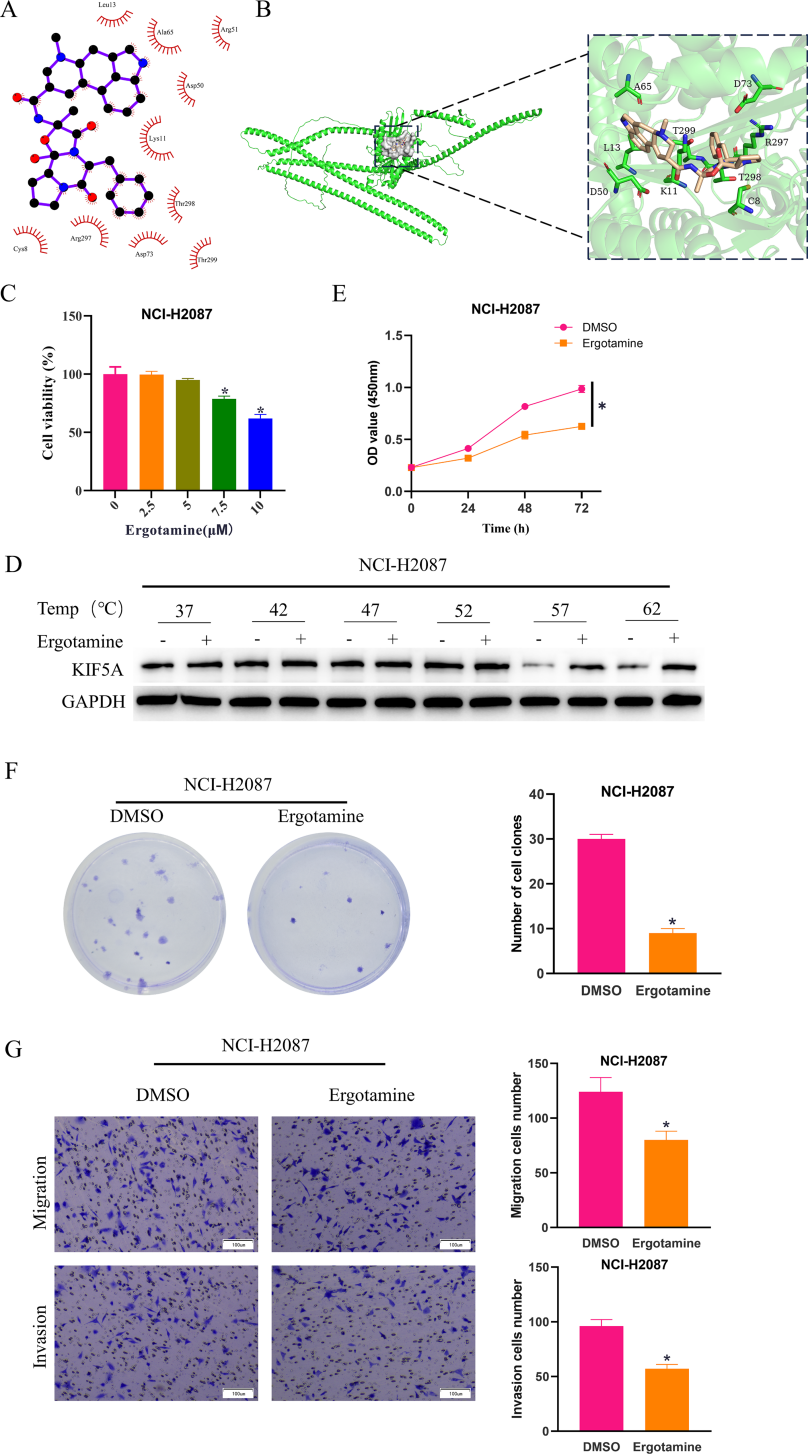
The binding relationship between Ergotamine and KIF5A and its effect on anoikis of LUAD cells. (A,B) 2D (A) and 3D (B) diagrams of Ergotamine targeting KIF5A binding sites. (C) CCK‐8 assessed cell viability after treatment with gradient concentrations of Ergotamine in NCI‐H2087 cells. (D) CETSA‐WB verified the binding relationship between Ergotamine and KIF5A. (E) CCK‐8 measured cell viability in NCI‐H2087 cells treated with DMSO or Ergotamine. (F) Cell colony formation experiment detected the proliferation ability of NCI‐H2087 cells treated with DMSO or Ergotamine. (G) Transwell assay measured the migration and invasion abilities of NCI‐H2087 cells treated with DMSO or Ergotamine. * means *p* < 0.05.

With FDA‐approved small molecules as ligands, batch molecular docking of target genes was carried out to screen out the small molecular drug Ergotamine with an optimal affinity score to KIF5A. The 2D (Figure 3A) and 3D (Figure 3B) diagrams of hydrogen bonding and hydrophobic interactions were plotted. Next, we treated NCI‐H2087 cells with gradient concentrations of Ergotamine (0, 2.5, 5, 7.5, 10 μM) and measured cell viability at different concentrations using CCK‐8, unearthing that Ergotamine exhibited dose‐dependent repression on cell viability when its concentration surpassed 5 μM, with the most pronounced repression observed at 10 μM. Therefore, we chose this concentration for subsequent experiments (Figure 3C). To further validate the binding relationship between Ergotamine and KIF5A, we also launched CETSA, which demonstrated that compared to the control group, Ergotamine treatment remarkably reinforced the thermal stability of KIF5A at 57°C and 62°C, confirming the binding relationship between the two (Figure 3D). Next, we set up two groups (DMSO and Ergotamine) based on NCI‐H2087 cells and seeded the two groups of cells on ultralow attachment plates to assess the effect of Ergotamine on LUAD cells anoikis. First, the viability of NCI‐H2087 cells in each group was detected by CCK‐8, which revealed that compared to the control group, the cell viability in the Ergotamine group was considerably reduced (Figure 3E). According to the cell colony formation experiment, Ergotamine treatment reduced the number of NCI‐H2087 cell colonies and repressed the proliferation ability of cells (Figure 3F). In addition, Transwell assay revealed that Ergotamine treatment reduced the number of migrating and invading NCI‐H2087 cells and inhibited the migratory and invasive abilities of the cells (Figure 3G). Given the above results, Ergotamine can bind to KIF5A and facilitate the anoikis of LUAD cells.

### 3.4 Ergotamine Targets KIF5A to Reinforce Anoikis in LUAD

**FIGURE 4:**
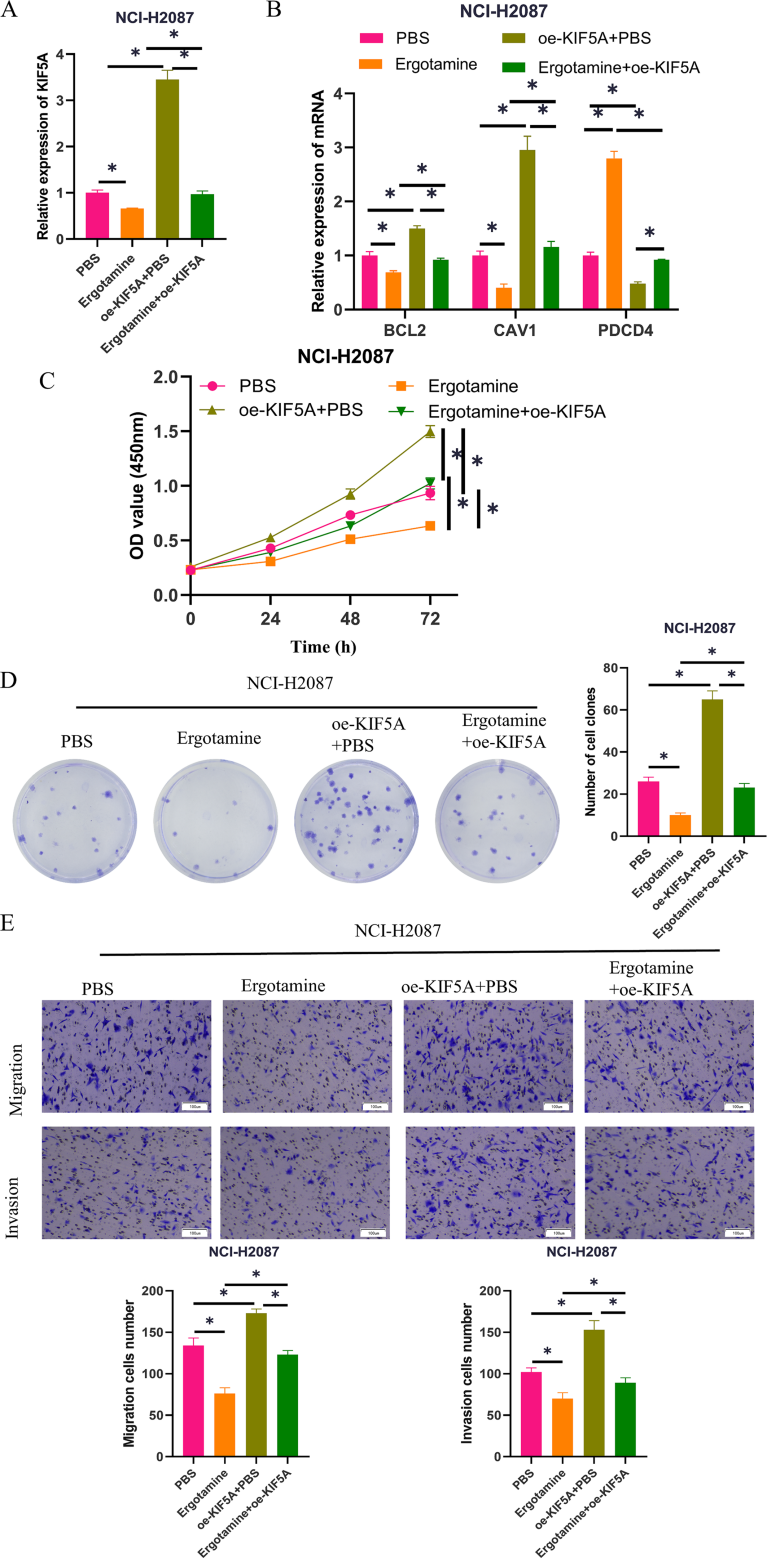
The effect of Ergotamine targeting KIF5A on anoikis of LUAD cells. (A) qPCR detected KIF5A expression in different groups of NCI‐H2087 cells; (B) qPCR measured the expression levels of BCL2 and CAV1 genes as well as PDCD4 gene in NCI‐H2087 cells. (C) CCK‐8 detected the viability of NCI‐H2087 cells in different groups. (D) Cell colony formation experiment determined the proliferation ability of NCI‐H2087 cells in different treatment groups. (E) Transwell assay determined the migration and invasion abilities of NCI‐H2087 cells in different treatment groups. * means *p* < 0.05.

Our experimental results verified the binding relationship between Ergotamine and KIF5A as well as the promoting role of Ergotamine on LUAD anoikis. Therefore, we speculated that Ergotamine can facilitate LUAD anoikis by targeting and modulating KIF5A expression. To verify this hypothesis, we constructed the following groups based on NCI‐H2087 cells:PBS, Ergotamine, oe‐KIF5A + PBS, and Ergotamine+oe‐KIF5A. First, we detected the expression of KIF5A in NCI‐H2087 cells from different groups by qPCR, finding that compared to the control group, the addition of Ergotamine alone greatly repressed the expression of KIF5A, which was restored to the control level by overexpression of KIF5A (Figure 4A). Subsequent qPCR analyzed the expression levels of anoikis‐repressing genes (BCL2 and CAV1) and the anoikis‐inducing gene PDCD4 in LUAD cells, revealing that in NCI‐H2087 cells treated with Ergotamine, the expression levels of BCL2 and CAV1 were decreased. In contrast, the expression level of PDCD4 was increased. However, overexpression of KIF5A reversed the effects conferred by Ergotamine treatment on the key anoikis‐related genes BCL2, CAV1, and PDCD4 (Figure 4B). CCK‐8 results demonstrated that the addition of Ergotamine alone decreased the viability of NCI‐H2087 cells, which was reversed to the control group level when KIF5A was overexpressed simultaneously (Figure 4C). Cell colony formation experiments were employed to evaluate the proliferation ability of NCI‐H2087 cells in different treatment groups, uncovering that compared to the control group, the proliferation ability of tumor cells in the Ergotamine treatment group was considerably reduced, which was reversed by KIF5A overexpression (Figure 4D). Meanwhile, by Transwell assay, it was found that Ergotamine treatment alone significantly reduced the number of migrating and invading cells compared with the control group, while overexpression of KIF5A at the same time brought the number of migrating and invading cells back to the level of the control group (Figure 4E). Collectively, Ergotamine facilitates LUAD anoikis by repressing KIF5A expression.

## 4 Discussion

In this project, we investigated the impact of KIF5A on anoikis of LUAD, identified the small molecular drug Ergotamine that possessed optimal affinity with KIF5A, and demonstrated that Ergotamine can target and repress KIF5A expression to trigger anoikis of LUAD cells (Figure S1).

Kinesin is implicated in multiple key biological processes including cell division and is tightly linked to cell proliferation, differentiation, and cell cycle progression [29]. Mounting evidence suggested that kinesin plays a pivotal part in cancer occurrence and progression [30]. As one of the members of the kinesin superfamily, KIF5A may also be related to cancer progression. Based on this, we first analyzed the expression of KIF5A in LUAD, finding that KIF5A was greatly upregulated in both tumor tissues and cells, which is in line with the results observed by Dong et al. [31] in LUAD. In addition, Dong et al. [31] also discovered that KIF5A can promote docetaxel resistance in LUAD. LC is the leading cause of cancer death, and exploring LC markers has important prognostic implications. Currently, both p16 and glucose transporter type 1 (GLUT1) have been shown to be overexpressed in malignant LCs, which are potential prognostic factors for new target therapy [32]. Our further survival analysis showed that the abnormally high expression of KIF5A in LUAD may be associated with poor prognosis of patients. This is consistent with the findings of Liu et al. who found that KIF5A was abnormally upregulated in hepatocellular carcinoma (LIHC), and its high expression level may be positively correlated with the malignant phenotype of LIHC, suggesting that KIF5A may be associated with LIHC disease progression [33]. In addition, most proteins in the kinesin superfamily, including KIF5A, have been proven to reinforce cancer cell metastasis [24, 34, 35]. A large number of recent studies have elucidated that cancer cell metastasis requires overcoming the cell programmatic death pathway of anoikis. Interestingly, we discovered that KIF5A was enriched in the anoikis pathway through the bioinformatics analysis approach. Currently, there are no studies about the impact of KIF5A on anoikis and its underlying mechanisms in LUAD. Subsequently, we cultivated LUAD cells on ultralow attachment plates to simulate the suspended state of anoikis in vitro in LUAD cells and dissected the effect of KIF5A knockdown on anoikis of LUAD cells. Herein, we proved that KIF5A was an inhibitory factor for anoikis in LUAD cells. Knocking down KIF5A facilitated the occurrence of anoikis in LUAD cells. Overall, our research indicated that KIF5A is essential in the anoikis of LUAD. Targeting KIF5A may bring new hope for LUAD patients.

We also screened out a small molecular drug Ergotamine with a strong binding ability to KIF5A through molecular docking experiments and confirmed their binding relationship through CETSA. Ergotamine is an ergot alkaloid that is present in ergot bacteria, which can be widely applied to the treatment of acute attacks of migraine and the prevention of postpartum hemorrhages. Ergotamine has been proven to repress tumor growth as early as 1980 [36]. By drug repositioning and molecular dynamics simulations, a recent study pointed out that the compound ergotamine may become a reusable drug for treating recurrent thymic epithelial tumors (TET) by targeting hnRNPA2b1 [37]. Additionally, it is worth noting that Liu et al. [38] employed online pharmacology, liquid chromatography, and the Lewis LC mouse model to identify Ergotamine as one of the key active ingredients in the traditional Chinese medicine prescription Yin–Huo–Tang (YHT) to prevent LUAD recurrence. These previous conclusions all indicated the application value of Ergotamine in the field of antitumor therapy. Similarly, in this investigation, we observed that Ergotamine reinforced LUAD cell anoikis and repressed cell proliferation. Furthermore, Ergotamine also targeted the inhibition of KIF5A to facilitate LUAD anoikis, which has not been reported in previous studies. Specifically, although high expression of KIF5A in LUAD promotes cancer progression, inhibits anoikis, under Ergotamine treatment, the expression of the pro‐cancer gene KIF5A is suppressed, leading to anoikis of the cells, which may play a certain anti‐tumor role. In conclusion, it suggested that the small molecular drug Ergotamine may be utilized as an anticancer agent for LUAD treatment.

In summary, our project proposed a new mechanism, revealing that Ergotamine targets KIF5A and represses its expression, thereby inducing anoikis of LUAD cells. These findings offer a new option for LUAD treatment based on targeting anoikis, providing a basis for the application of the small molecular drug Ergotamine. However, our work was conducted based on cell experiments, lacking animal models to verify its authenticity. Therefore, further confirmation is required in future experiments.

## Author Contributions

Bin Bao conceived of the study and participated in its design and interpretation and helped to draft the manuscript. Xiaojun Yu and Wujun Zheng participated in the design and interpretation of the data and drafting/revising the manuscript. Jiewei Sun performed the statistical analysis and revised the manuscript critically. All the authors read and approved the final manuscript.

## Ethics Statement

Ethical approval is not required for this study in accordance with local or national guidelines.

## Consent

Patient consent were not required in accordance with local or national guidelines.

## Conflicts of Interest

The authors declare no conflicts of interest.

## Supporting information


**Figure S1** Graphic abstract of LUAD cell regulatory mechanisms. KIF5A inhibits LUAD cell anoikis, while the small molecule drug Ergotamine targets and inhibits the expression of KIF5A, which in turn induces LUAD cell anoikis.

**Table S1** Supplementary Information.

## Data Availability

The data and materials in the current study are available from the corresponding author on reasonable request.
