# Supplementary figures and images for: Ergotamine Targets KIF5A to Facilitate Anoikis in Lung Adenocarcinoma
Source: Clin Respir J. 2024 Nov 8;18(11):e70020. doi: 10.1111/crj.70020 (PMC11549061; doi:10.1111/crj.70020)

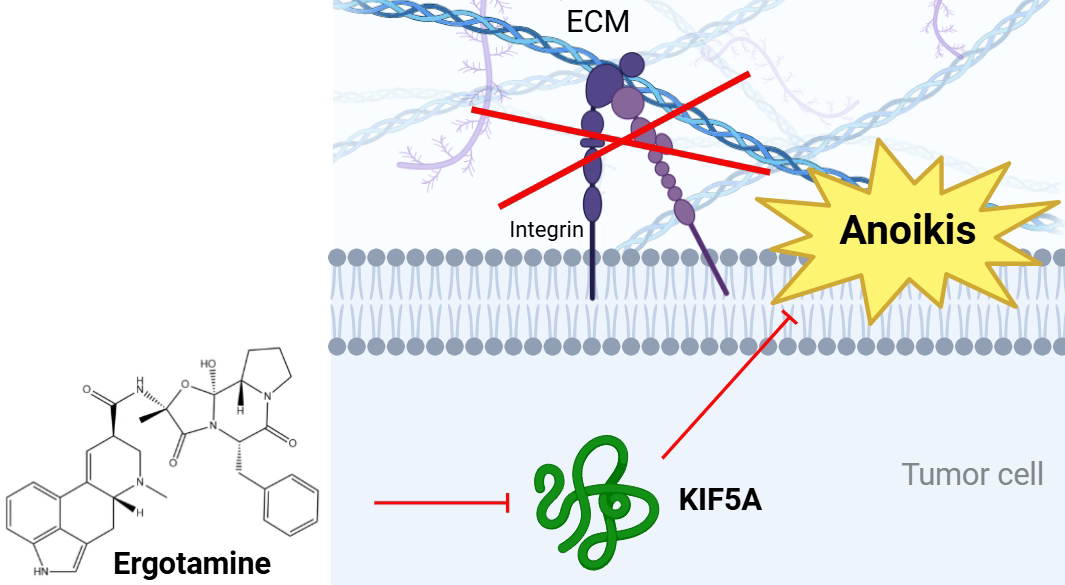

Supplement: Supplementary file 1 — Figure S1 Graphic abstract of LUAD cell regulatory mechanisms. KIF5A inhibits LUAD cell anoikis, while the small molecule drug Ergotamine targets and inhibits the expression of KIF5A, which in turn induces LUAD cell anoikis. [file CRJ-18-e70020-s001.png]
